# Exploring a faith-based approach to puberty education in Tanzania

**DOI:** 10.3389/frph.2023.1024550

**Published:** 2023-02-10

**Authors:** Hawi Teizazu, Caitlin Gruer, Elisia Mandara, Marni Sommer

**Affiliations:** Department of Sociomedical Sciences, Mailman School of Public Health, Columbia University, New York, NY, United States

**Keywords:** adolescents, health, faith-Based organisations (FBOs), gender, Tanzania

## Abstract

**Background:**

Many adolescents in Tanzania do not receive timely and comprehensive puberty education. This study explored faith-based organizations a site for puberty education. Two puberty books, each developed through participatory research with Tanzanian adolescents and stakeholders, were promoted to 177 Christian denomination churches in Dar es Salaam, Tanzania to understand the factors that faith leaders considered in their decision to purchase puberty books, or share information about the intervention to their peers and congregants.

**Methods:**

Data collection included routine monitoring *via* weekly reports and ethnographic observation. Data were analyzed using the Ecological Framework for Health Promotion to capture how individual, interpersonal, and institutional factors influenced leaders' decisions to purchase or promote puberty books.

**Results:**

At the individual level, leaders cited their personal experiences in their support for the intervention, but leaders' time and confidence in their ability to effectively promote books to others were barriers to participation. Interpersonally, the diffusion of information between church leaders, particularly when information came from well-known or respected leaders, emerged as an important factor in leaders' willingness to promote books. At the institutional level, leaders' decisions were impacted by resources, institutional culture, and institutional hierarchy. Importantly, twelve churches in the sample purchased books. Limited financial resources and the need to receive approval from denominational leaders were discussed by leaders as barriers to purchasing books.

**Conclusions:**

Despite research showing high religiosity in Tanzania, the role of religious institutions in providing puberty education has remained unexplored. Our results inform future research and practice by providing an articulation of the socioecological factors that played a role in faith leaders' decisions related to puberty education interventions in Tanzania.

## Introduction

Puberty is an important, but often neglected, aspect of reproductive health. The guidance received during this critical stage informs how individuals navigate their reproductive health decisions throughout the lifespan. Although research indicates the importance of puberty education, many young people undergo bodily changes without adequate guidance or support. Furthermore, health efforts to address this education gap have primarily been directed towards schools, despite the preeminent role of social institutions in educating young people internationally. This study seeks to explore faith-based organizations (FBOs) as a site for puberty education in Tanzania by introducing culturally-informed and evidence-based puberty books to the leaders of Christian FBOs in Dar es Salaam.

There is growing global attention paid to early adolescence as a critical stage in the transition to adulthood (1–3). Young people are the world's fastest growing population group, which includes over 1.2 billion adolescents aged 10–19, the majority living in low-income countries (4). Tanzania, the site of this study, is home to 12 million adolescents (5). One aspect of this growing global attention focuses specifically on the onset of menstruation among girls in low-income countries and the inadequate guidance and support received about sexual and reproductive health (1, 6, 7). The onset of menstruation is an opportunity to reach girls, their parents, caregivers, and teachers, and a stepping stone to talking about topics such as sexual behaviors and contraception when they get older (3, 7). Similarly, studies suggest that boys also receive inadequate guidance on pubertal body changes, and healthy ways to cope with intensifying peer and societal pressures that arise during puberty (8–12). This lack of guidance has clear implications for population health and gender equity, as it impacts knowledge and awareness about diseases such as HIV and AIDS, health behaviors such as contraceptive use and age at first sex, and attitudes about gender norms and gender-based violence (13).

In Tanzania, specifically, puberty education is an unfulfilled health need. Research points to significant gaps in the puberty-related guidance that adolescents receive in Tanzania (8, 14). Most school-based sexual and reproductive health education in Tanzania occurs during secondary school (ages 14–17) and focuses primarily on HIV and AIDS (15, 16). As most girls reach puberty between the ages of 8 and 13, and boys between the ages of 9 and 14 (17), the absence of puberty education during primary school (ages 7–13), when bodies begin to change, may lead to fear and confusion. Additionally, Tanzanian parents may be more amenable to adolescents learning about pubertal changes in advance of broader lessons on sexual and reproductive health, which is an uncomfortable topic for many parents (8, 9). Notably, the education provided in schools fails to reach many of Tanzania's adolescents, as over 40 percent do not attend secondary school (18). For these reasons, utilizing social and cultural institutions, such as faith-based organizations, for puberty education interventions may substantially improve outreach.

In addition to reaching adolescents in Tanzania that do not receive puberty education in schools, our exploration of religious institutions as a site for puberty education recognizes the findings of surveys, which point to high levels of religiosity among Tanzania's population. Over 98% of Tanzanians report a religious affiliation, primarily Christianity (61.4%) and Islam (35.2%). Roman Catholics and Lutherans represent the majority of Christians, and the Evangelical Lutheran Church in Tanzania represents one of the largest Lutheran denominations in the world (19, 20). Previous public health efforts have recognized the notable role of FBOs as cultural institutions across different communities, and have often turned to faith-based organizations throughout the African continent to support their efforts to improve health outcomes. These include efforts to reduce stigma around HIV testing and treatment (21). More specific to the study described here, faith-based approaches have been utilized to distribute puberty content to adolescents throughout sub-Saharan Africa. In 2005, *Strategies for Hope* developed the “Called to Care” toolkit, a Christian-inspired HIV education intervention for faith-based organizations in sub-Saharan Africa. Age-appropriate books were shared with local FBOs, including churches, with information on peer pressure, changing bodies, and Bible study. Although it is unknown how books were used by FBOs, demand for the books outpaced supply and funding (22).

To provide a culturally-tailored and evidence-based puberty education for Tanzanian youth, a Tanzanian girl's puberty book was developed in 2009 to provide guidance during the transition from childhood into adolescence and young adulthood (23). The book was approved by the Tanzanian Ministry of Education, along with a book developed for boys in 2012 to address boys' similar lack of guidance (8). Development for both books began by engaging key stakeholders in the country, such as the Ministry of Education, adolescent health experts, and actors working at United Nations (UN) agencies and non-governmental organizations (NGOs). Second, participatory research with adolescent girls and boys, respectively, captured lived experiences about first periods, wet dreams, peer pressures, myths about body changes, and questions about their changing bodies. The books were field-tested with youth (10–14 years), teachers, parents, and key stakeholders to assure reading comprehension and content appropriateness. The books were printed by a local publisher in Tanzania with copies ordered by the government, global UN agencies, and local and international NGOs. To date over 430,000 girls' books and over 250,000 boy's books have been distributed across Tanzania. An evaluation of a similarly designed girl's puberty book in Ethiopia found that reading the book increased girls' levels of knowledge around menstruation and puberty (6). Considering the demonstrated need for puberty education and the success of culturally-informed puberty books, exploring the feasibility of incorporating puberty books into new institutions in a sustainable way is essential for reaching additional adolescents in Tanzania.

This study provides a necessary exploration of faith-based organizations as a site for puberty education, specifically. Previous research has examined the factors impacting the uptake of health interventions related to HIV education (24, 25), infant and maternal health (26), and family planning (27) within FBOs in Tanzania, but no study to date has examined the delivery of puberty education within Tanzanian FBOs. In this novel effort to incorporate sustainable puberty book distribution within faith-based organizations (FBOs) in Tanzania, we examined the different factors that church leaders considered in their decision-making around promoting puberty books. For the purpose of this study, puberty book promotion was defined as directly purchasing books and distributing them to congregants, encouraging congregants to obtain the book(s) from local distributors, and sharing information about the books to individuals within their networks. Because FBOs operate with a distinct set of norms and practices, it was important to identify the factors that leaders considered before not only purchasing books, but also before choosing to promote books within this specific cultural context.

This study's identification of the different factors that influenced the decisions of faith leaders was guided by the Ecological Model for Health Promotion created by McLeroy and colleagues (28), which recognizes the social and organizational influences on individual health behavior and decision-making. Specifically, the framework understands individual behavior as being subject to influences at five different ecological levels: individual characteristics (intrapersonal), social network (interpersonal), the rules and characteristics of the institutions to which they belong (institutional), the relationships between organizations and networks (community), and the policies that govern action within a defined boundary (public policy). The use of the ecological framework as a conceptual guide allows for a recognition of faith leaders' agency when making decisions related to the operations of their FBO, as well as a recognition of how their decisions are impacted by their personal resources and experiences, the influence of their peers, and the informal and formal rules within this religious setting. By applying an ecological perspective to analyze weekly reports that detailed interactions with FBO leaders in Dar es Salaam, we identified the multi-level factors that leaders considered in their decision to purchase or promote puberty books in order to inform future efforts to introduce similar material to FBOs.

## Materials and methods

### Setting and ethics

Dar es Salaam was selected to explore the potential uptake of puberty books by FBOs because it is the largest city in Tanzania (29) and has a high concentration of well-attended FBOs (30). It was assumed that FBOs in Dar es Salaam may be well-resourced and therefore more receptive to the intervention, given their congregation sizes and proximity to financial centers. Lessons learned could be subsequently applied to efforts elsewhere in Tanzania. This study received Internal Review Board approval from the Columbia University Medical Center Institutional Review Board (CUMC IRB)(AAAS6265) and the Tanzania Commission for Science and Technology (COSTECH)(No.2020-063-NA-2019-395).

### Training and study design

Outreach to FBOs was conducted by a staff member based in Dar es Salaam, who was trained by two of the study's authors about the puberty books, the process of developing the books, and project tracking tools. The project was implemented from October 2019–March 2021, with three months of interruption due to COVID-19.

### Sampling

Churches were purposively selected to ensure diversity in terms of denomination, location and congregation size. An initial desk review generated a list of churches from dominant and smaller denominations in Dar es Salaam, along with coordinating and leadership bodies for both Christians and Muslims. Subsequent stakeholder consultation identified large FBOs within each district of the city. A decision was made to initially focus on various denominations of Christian churches because the Muslim leadership council overseeing many mosques in Dar es Salaam requested a formal internal review of the book that would have extended beyond the study timeline. After mapping, the field-based staff member spent sixteen months introducing the puberty books (in person or *via* phone) to churches (*n* = 177) in targeted districts of Dar es Salaam. If the church leader expressed interest in the books, the staff member followed up at least three times through in-person visits or by phone.

### Ethnographic observation and routine monitoring

The data for this study came from weekly monitoring reports and ethnographic observations. Weekly reports included documentation of activities and informal conversations, and routine tracking of the introduction, purchase, and distribution of the puberty books. The reports also included the following information for each visit and call with FBO leaders: the name and denomination of the FBO, a description of what occurred, the stakeholders involved in the interaction, and information shared by leaders. Ethnographic data consisted of field notes that captured observations made when meeting with church leaders, attending church events, and attending Sunday services or meetings between leaders, when invited. Weekly reports and field notes were completed by one individual, which strengthened consistency in the process and comparability across reports. Reports were reviewed weekly by two CU researchers and any questions or clarifications were discussed with the staff member and updated in the report.

### Data analysis

Analysis began by reading weekly reports and transferring the information to a data charting matrix. The matrix was stratified by FBO, and allowed for the identification of patterns in the events across FBOs, as well as an understanding of the progression of events within each FBO over time. To analyze the data, we referenced the Ecological Model for Health Promotion as an analytical guide (28) to contextualize individuals' health-related decisions within individual and environmental factors. Using this model as a framework, we examined the data and noted the factors at the individual, interpersonal, and organizational levels that emerged in ethnographic notes and in conversations with FBO leaders. These factors were identified and defined iteratively, following the process of thematic analysis used in qualitative research. In this process, the data matrix was first read by each team member to establish familiarity with the data. After repeated readings of the data, the research team, including the on-site staff member, met to discuss emergent meanings and patterns and operationalize them using an initial set of codes. The codes were defined and tested using a subsample of the data. The research team met to discuss refinements and create a final set of codes which were subsequently applied to the complete data set. The research team met at the midpoint and end of the analysis process to establish and affirm coding agreement. The codes were grouped into themes, and the research team met to determine their conceptual boundaries and decide which themes should be collapsed. The final themes were classified as factors under the levels of the ecological model, and summarized through a narrative description, as well as through tables that contained definitions, examples, and how themes factored into leaders' decision-making.

## Results

Of the 177 churches approached during the study period, twelve directly purchased books. The purchase order size ranged from 10 to 200 books, with an even number of girls' and boys’ puberty books. Books were purchased by the following denominations: Evangelical Lutheran Church of Tanzania (*n* = 5), Tanzania Assemblies of God (TAG) (*n* = 3), Roman Catholic (*n* = 1), Anglican (*n* = 1), Mennonite (*n* = 1) and Methodist (*n* = 1). There were several factors at the individual, interpersonal, and institutional level that played a role in the uptake and promotion of books by the church leaders included in the study.

### Individual

Individual level factors were characteristics specific to individual leaders that emerged as considerable factors in their decision making related to book promotion (Table 1). These factors included leaders’ personal experiences, their time, and leaders' self-concept. Leaders referenced personal experiences, which included their memories of adolescence or their experiences as parents of adolescents, when discussing the need for puberty books within their institution. Time was discussed by many leaders who chose not to promote books. Leaders, even those who personally endorsed the intervention, pointed to existing demands on their time and were hesitant to promote books within their institution. Importantly, in all but two of the FBOs that purchased books, lead pastors were able to delegate the task to internal committees that focused on the wellbeing of youth and adolescents, or congregants more broadly. Lastly, leaders' self-concept emerged as an important factor, as leaders who were confident in their ability to persuade others in their network were more willing to promote books.

**Table 1:** The impact of individual-level factors on church leaders' promotion of puberty books.

Factor	Description	Examples	Role in leaders’ promotion of the intervention
Personal experiences	Past or current experiences that impact how leaders perceive the intervention	Relevant personal experiences included leaders’ experiences during puberty (e.g., feeling uninformed, confused), role as teachers or working with youth, and their experiences as parents to adolescents	Leaders’ personal experiences positively influenced their support of the intervention.
Time	Leaders’ ability or inability to devote time to learning about the books, discussing books with other leaders, and actively promoting books to congregants	Demands on leaders’ time included serving in multiple leadership roles, travel, and tending to congregant needs	Leaders viewed time as a barrier to participation. Leaders could not find time to discuss books with staff, and declined participating due to the time it would take to promote books to congregants.
Self-concept	How leaders perceived their ability to effectively promote books to peers and congregants	Expressing hesitation to approach leadership or peers, solicitating assistance from research team members when sharing information about books to peers or congregants.	Low confidence in one's ability to purchase or promote books was a barrier to engagement, and affected if and how leaders shared information within their networks.

### Interpersonal

Two interpersonal factors, network integration and social status, captured the ways that leaders' social networks played a role in the uptake and promotion of puberty books. (Table 2). Church leaders who were highly integrated within their social and professional networks had more opportunities to share or hear information about the intervention. This diffusion of information occurred during routine meetings among church leaders and when leaders visited services in other churches where books were being sold. Notably, leaders of Lutheran churches cited processes of diffusion more than leaders of other denominations in the study. In three of the five Lutheran churches that purchased books, leaders had heard about the intervention through their peers at regional meetings or Bible studies with leaders of neighboring FBOs. Social status emerged as the second interpersonal factor. Leaders who learned that the intervention was endorsed by influential leaders in their networks expressed positive attitudes towards book uptake and promotion. Influential leaders included formal and informal leaders who were well-regarded among peers and congregants as well as leaders who presided over multiple FBOs.

**Table 2:** The impact of interpersonal-level factors on church leaders' promotion of puberty books.

Factor	Description	Examples	Role in leaders’ promotion of the intervention
Network integration	The extent to which leaders were enmeshed in their social or professional networks	Leaders who were highly integrated in their networks discussed attending Bible studies, regional meetings where information about books was being shared, and other churches where books were being presented.	Being integrated into social and professional networks factored positively in book promotion, as it allowed leaders to have frequent opportunities to share or receive information about books.
Social status	Individuals with considerable influence over the actions of peers and/or congregants.	Leaders who had favorable reputations, and were well-liked and respected among peers and congregants.	Influential leaders were important agents of social mobilization. The opinions of influential regional leaders shaped wider perceptions of the intervention among peers and congregants.

### Institutional

At the institutional level, three factors emerged as important considerations in leaders' decision-making: resources, institutional hierarchy, and culture (Table 3). All of the leaders who declined purchasing books discussed limited financial resources, and some pointed to the effects of COVID-19 when discussing the need to focus on core expenditures (e.g., salaries, building costs), rather than engage in new projects such as book promotion. Limited financial resources were also discussed by some leaders who chose to purchase books, but because FBOs could set their own prices for the books, these leaders sold puberty books at a profit to fundraise for other projects within their FBO. Leaders of well-resourced FBOs could distribute books to congregants at no cost. Secondly, leaders discussed how formalized systems of hierarchy within their institutions posed a challenge to participation. Before purchasing or promoting books, leaders communicated the need to receive formal approval from leaders within their respective institutions as well as leaders within the hierarchical structure of their denomination.

**Table 3:** The impact of institutional-level factors on church leaders' promotion of puberty books.

Factor	Description	Examples	Role in leaders’ promotion of the intervention
Resources	The ability to purchase and sell books, as indicated by a church's financial resources.	Financial resources included the funds available within the FBO and the financial capacity of congregants	Limited resources restricted the capacity of otherwise interested leaders from adopting the intervention.
Hierarchy	Formalized systems of ranked authority through which leaders’ activities related to the intervention needed to be approved	Each FBO had an internal hierarchical system (e.g., senior pastors, assistant pastors), and FBOs were integrated into broader denominational hierarchies	Hierarchy was discussed as a barrier to leaders’ participation. Leaders often needed to receive approval from senior pastors/Bishops within their institution, as well as denominational leadership, before participation.
Institutional culture	Values and customary practices within FBOs	Cultural factors described by church leaders included religious beliefs, and norms around engaging in nonreligious activities.	Leaders were more willing to participate if they perceived the books as aligning with their institution's culture, or could identify ways to incorporate puberty books into existing practices in their FBO.

Lastly, leaders described aspects related to institutional culture when discussing book uptake and promotion. Before promoting books, leaders assessed whether the puberty books aligned with their institution's values. This included ensuring that the language and images aligned with religious values concerning sexual behavior. In addition to values, leaders discussed informal institutional norms that influenced their decision-making. Leaders declined to participate if the act of selling or promoting non-religious material, or advocating for nonreligious issues, was uncommon in their FBO. Other leaders discussed how puberty books could be incorporated into existing norms within their institutions. This included distributing books as a complement to religious teaching directed towards adolescents, and incorporating books into significant cultural events within the FBO (e.g., first communion, Christmas celebrations, and dedication or confirmation services).

## Discussion

Overall, leaders indicated a strong need for puberty education for youth. Among leaders who decided to purchase books, the majority led Lutheran FBOs. Previous research has found denominational differences in the uptake and implementation of faith-based interventions (31), but has also recognized heterogeneity within denominations, highlighting the integral role of local leaders (32). The denominational differences observed in this study may have been due to patterned differences in the interpersonal or institutional factors that were shown to impact leaders' decision making, but could have also been due to individual-level differences between leaders.

Nearly all leaders in the study recognized the need for puberty education, and discussed how their own personal experiences during adolescence, working with adolescents, or parenting adolescents facilitated an awareness of the absence of education around this issue. Previous studies that examined the role of church leaders in faith-based health interventions have also found that leaders were more likely to endorse interventions when they had personal ties to members of the affected population and familiarity with the issue targeted by the intervention (33–35). Importantly, this recognition was an insufficient prerequisite for uptake, as indicated by the low number of institutions that purchased books. The majority of leaders who did not purchase books supported the idea that FBOs should play an active role in puberty education, but described several barriers to purchasing or promoting books. Time emerged as universal barriers for leaders. Demands on leaders' time has been recognized in studies examining the barriers to faith-based health interventions to address HIV (33, 35–37), even among leaders who perceived the intervention favorably(35, 36).

Furthermore, institutional resources and institutional hierarchy were often discussed by leaders as a barrier to engagement. Resources were a profoundly impactful barrier to purchasing books. Although the cost to the institution and the cost to congregants were discussed separately by church leaders, FBOs and their congregants were financially-linked through tithes and offerings, meaning that well-resourced churches often had higher-income congregants. Finances have long been recognized as barriers to health program implementation (38). Importantly, limited resources did not preclude leaders from purchasing books. Leaders of under-resourced FBOs strategically sold books to generate funds for projects (e.g., building maintenance or expansion). Previous research has found that financial incentives in faith-based health interventions have served to meet the competing financial demands faced by leaders of FBOs (39).

Institutional hierarchy also restricted leaders' ability to directly purchase books. All FBOs that purchased books required approval from lead pastors, who had the greatest authority within a given church. Additionally, the majority of the churches that purchased books belonged to denominations in which the study's investigators had preemptively received approval from overarching leadership. Previous studies on health interventions in FBOs indicate that hierarchical approval is a prerequisite, particularly when addressing sensitive issues. In their study of the factors impacting the uptake of HIV interventions in FBOs in Tanzania, Hartwig and colleagues (24) found that interventions were opposed by senior pastors in the five churches in their sample that lacked HIV education efforts.

The ability of leaders to effectively promote books to their peers and congregants was related to their involvement within existing social networks, and their status within these networks. Information about the books spread intra-denominationally through recurring local meetings, attendance at local churches, and regional meetings between overarching denominational leaders and pastors. Leaders were more willing to promote puberty books within their spheres of influence after hearing about books through network ties, especially if the information came from leaders with high social status. This finding aligned with previous research that has shown that health interventions in FBOs were perceived more favorably when they were endorsed by influential leaders (40), particularly leaders who took personal initiative in direct promotion and advocacy (33, 35). The role of influential leaders in this study, and in other studies, points to the need for future health promotion efforts to identify influential advocates within FBOs, as they have the potential to influence popular opinion within their spheres.

Lastly, it is important to discuss the cultural factors pertaining to this study's setting, primarily the ways that religion factored into leaders' decision making related to puberty content. The majority of leaders did not see religious customs and teachings as a barrier to puberty book promotion and distribution, but some leaders who declined distributing books pointed to how distributing nonreligious material would deviate from norms within their institution. These findings showed how institutional culture could constrain the actions of religious leaders, and is supported by existing research that has highlighted the role of leaders in actively shaping the culture of their institutions. A review examining the role of clergy in faith-based health interventions in the USA found them to be important mediators in the uptake of health promotion interventions in faith-based organizations (41). Through interviews with Tanzanian church leaders, Hartwig and colleagues (24) found that lead pastors had considerable influence on the attitudes of church members towards HIV. Additionally, research has found that interventions within FBOs are often adapted by leaders to suit a local context, and leaders communicate interventions to complement the religious values of each church (42, 43). A similar finding emerged in this study, as some leaders strategized ways to frame the puberty books in ways that were aligned with existing norms and practices in their FBO.

### Limitations and future research

Having one on-site staffer may have limited the intervention's reach, given the need for repeated visits to FBOs. Importantly, the COVID-19 pandemic led to the temporary closure of religious gatherings and youth-related programming, and diminished church attendance for 11 months during the 18-month study period. COVID-19 protocols were developed for the study, which included halting outreach for three months and implementing phone-based follow-up activities. While these impacts were unavoidable, they likely impacted the leaders' uptake of the intervention. Additionally, although this study was well-equipped to identify the multilevel factors that were considered by leaders in their decision-making process regarding book promotion and purchase, this study was limited in its ability to identify the factors that facilitated the purchase of books due to the small number of FBOs in the sample that agreed to purchase books.

Notably, this study was the first to explore the multilevel factors that impacted leaders' decision-making within FBOs in Dar es Salaam. These findings could inform future health promotion efforts and research in FBOs in this region. Of particular importance for health promotion initiatives was the role of key actors in our study, particularly those who were influential in terms of status or position in their institution's hierarchy, in promoting the intervention to peers. Future interventions could benefit from identifying influential individuals within FBO networks in order to positively influence local perception of the intervention. Additionally, the strategies used by leaders to overcome institutional-level barriers to book purchase and promotion (i.e., limited resources) could provide valuable guidance for future health interventions in FBOs in Dar es Salaam. Although this study did not seek to examine how denomination influenced participation, we found denominational differences in uptake, as the majority of churches that purchased books were from the Lutheran denomination. Future research should aim to explore the role of denomination through purposive sampling. Lastly, the findings of this study could not be generalized to Muslim institutions in Dar es Salaam, which had their own diversity of leaders and institutions. Future efforts should prioritize Muslim FBOs to improve outreach among additional groups in the Tanzanian population and to understand perceptions of puberty books among leaders of Muslim FBOs.

## Data Availability

The raw data supporting the conclusions of this article will be made available by the authors, without undue reservation.
